# Single cell whole-genome sequencing of brain cells: age- and cell-type specific mutational profiles

**DOI:** 10.1038/s41392-024-01892-3

**Published:** 2024-08-09

**Authors:** Melania Capasso, N. Ahmad Aziz

**Affiliations:** 1 Immune Regulation, German Center for Neurodegenerative Diseases (DZNE), Bonn, Germany; 2 Population and Clinical Neuroepidemiology, German Center for Neurodegenerative Diseases (DZNE), Bonn, Germany; 3 Department of Neurology, Faculty of Medicine, University of Bonn, Bonn, Germany

**Keywords:** Genetics of the nervous system, Neurological disorders

**Fig. 1:**
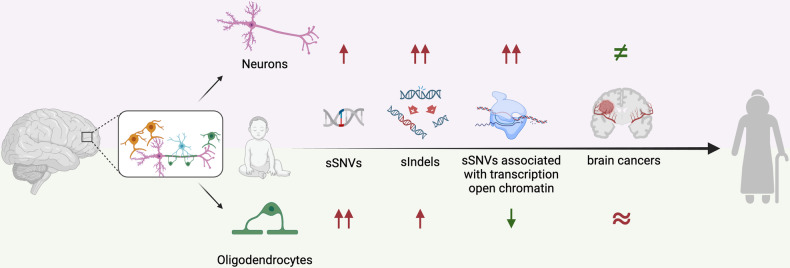
Schematic representation of findings by Ganz et al.,^1^ highlighting differences in somatic mutations between neurons and oligodendrocytes. sSNVs: somatic single nucleotide variants; sIndels: somatic insertions and deletions. Created with BioRender.com

In a recent study published in *Cell*, Ganz et al. shed new light on the mutational landscape of brain cells, particularly neurons and oligodendrocytes (OLs).^1^ Utilizing a combination of optimized single-cell whole-genome sequencing with single-nucleus chromatin accessibility and gene expression analysis, they profiled somatic mutations in 86 OLs from 13 neurotypical individuals, spanning in age from infants to elderly. Neurons investigated were 56, derived from 19 (including 12 overlapping) individuals (Fig. 1).

The brain comprises a diverse set of cell types, with high regional and functional specificity. Neurons have diverse phenotypes and are supported in their function by macroglia, including astrocytes and OLs, the latter producing myelin to ensheath long axons, and microglia, the immune resident macrophages. These cells have a distinct developmental origin and differ in their cell division potential. While the great majority of neurons differentiate during development and remain in a postmitotic state throughout lifespan, glial cells retain their renewal capacity. In particular, oligodendrocyte precursor cells (OPCs) possess substantial proliferative potential, which might account for their purported role in the pathogenesis of the most common form of adult brain cancer, glioblastoma multiforme.

The study by Ganz et al. reveals intriguing differences in the mutational profiles of neurons and OLs during lifespan.^1^ Through direct comparison with matched bulk DNA sequencing data, they found that, although the load of somatic single nucleotide variants (sSNVs) increases with age in both cell types, this rate is much faster in OLs (29/year *vs*. 16/year). Indeed, OLs had 54% more sSNVs at birth compared to neurons, although this finding did not reach statistical significance. Conversely, the mutational load of small insertions/deletions (indels; IDs) was similar in the two cell types at birth but increased faster with age in neurons (2.9/year *vs*. 2.1/year). Nonetheless, the most striking difference between the two cell types was the observation that OL sSNVs were enriched in intergenic regions and depleted in coding regions, whereas the opposite pattern was observed in neurons, with a ~2-fold higher rate of potentially deleterious IDs in neurons.

Interestingly, the pattern of sSNVs in OLs was more similar to that of hemopoietic stem precursor cells (HSPCs) than neurons. This could indicate that most OL mutations arise before differentiation, while the cells are still in their OPC state. But what is the nature of these mutations? The authors addressed this point through systematic exploration of mutational signatures, including single base substitution (SBS) and ID signatures. SBS signatures arise from recurring trinucleotide patterns of transition/transversion of SNVs and adjacent nucleotides, while ID signatures are based on the number and type of nucleotides affected, as well as the presence of regions with repetitive patterns or microhomology.^2^ The clock-like SBS5 signature, which increases with age and is independent of cell division, had the highest prevalence in both cell types, but accumulated at a higher rate in OLs than neurons. The signatures SBS1 and SBS32 were strongly represented in OLs, but almost absent in neurons. SBS1 is a signature associated with cell division and SBS32 is a C > T substitution that also characterizes HSPCs, both suggesting an origin in the ancestral OPCs. The only signature that accumulated at a higher rate in neurons was SBS16, a signature associated with transcription, confirming that neuronal mutations have a predilection for transcribed genomic regions.^3^ The analysis of IDs also indicated differential signatures, with some specifically enriched in neurons with age (ID4, associated with transcription) or in OLs, like ID9, which is also present in gliomas and other brain tumors.

The authors were also able to study three pairs of OLs, likely originating from the same common ancestral cell, as determined by their similar mutational profiles. Based on the number of shared sSNVs and the average rate of sSNV accumulation per year in OLs, they estimated that two of these pairs separated near birth and one around 12 years of age. The first two pairs showed a stronger SBS1 signature, while the last pair presented signatures more similar to aged OLs, suggesting that, in the first years of human life, OPCs accumulate many mutations that will be retained in OLs later in life. However, to confirm this observation, a direct sequencing of OPCs at different ages would be required.

Overall, the analysis of sSNV and ID patterns with age, and their integration with transcriptional data from single-nucleus RNA-seq and chromatin accessibility through single-nucleus ATAC-seq, confirmed their initial observation that mutations in OLs are enriched in closed “dormant” chromatin, while the opposite is true for neurons, which show an increased frequency of sSNVs and IDs in transcribed regions. Interestingly, OL mutations were enriched in late-replicating regions of the genome, which have a known tendency to accumulate more mutations than early-replicating ones. This finding may, at least partly, also account for the preferential accumulation of OL mutations in dormant chromatin regions, because these are mainly late-replicating. Importantly, as opposed to neuronal mutations, OL mutations had a similar distribution to those found in brain cancers, especially glioblastoma multiforme. These observations raise the interesting question of whether sSNVs in OLs arise during their OPC state while the chromatin is still open, but either are not repaired because they become “silent” again upon chromatin closure, or are due to a lack of DNA repair activity.^1^ However, later in life, when repression of dormant chromatin becomes dysregulated, these mutations could become detrimental, either giving rise to cancer or contributing to neurodegenerative diseases, in which myelin disfunction is increasingly shown to play a role.^4^ To this end, a study of OL mutations in patients with neurodegenerative diseases, and whether these are driven by genomic instability due to reactivation of retrotransposable elements,^5^ would be very informative. Moreover, the finding that neurons accumulate many mutations in coding regions without suffering apparent catastrophic consequences, raises the question of whether somatic mutations could serve a purpose in differentiating neuronal phenotypes, or even provide resilience to insults related to aging. Or maybe they remain below a threshold still compatible with neuronal survival, but could tip the balance towards neuronal degeneration in the face of other insults, such as protein misfolding.

In conclusion, this study substantially expands our understanding of the mutational landscape of aging brain cells and provides a solid foundation for future investigations involving more cells types (e.g., astrocytes and microglia) and across different stages of development, in larger groups of individuals. This could allow an even clearer picture to emerge, one that might take us closer to understanding critical changes leading to brain cancer and neurodegeneration.
